# Protein Aggregation Capture on Microparticles Enables Multipurpose Proteomics Sample Preparation*[Fn FN2]

**DOI:** 10.1074/mcp.TIR118.001270

**Published:** 2019-03-04

**Authors:** Tanveer S. Batth, Maxim A. X. Tollenaere, Patrick Rüther, Alba Gonzalez-Franquesa, Bhargav S. Prabhakar, Simon Bekker-Jensen, Atul S. Deshmukh, Jesper V. Olsen

**Affiliations:** From the ‡The Novo Nordisk Foundation Center for Protein Research, University of Copenhagen, Denmark;; §Cellular Stress Signaling Group, Center for Healthy Aging, University of Copenhagen, Denmark;; ¶The Novo Nordisk Foundation Center for Basic Metabolic Research, University of Copenhagen, Denmark

**Keywords:** Protein Denaturation*, Mass Spectrometry, Phosphoproteome, Secretome, Affinity proteomics, Automation, magnetic beads, microparticles, protein aggregation, sample preparation

## Abstract

Universal proteomics sample preparation is challenging because of the high heterogeneity of biological samples. Here we describe a novel mechanism that exploits the inherent instability of denatured proteins for nonspecific immobilization on microparticles by protein aggregation capture. To demonstrate the general applicability of this mechanism, we analyzed phosphoproteomes, tissue proteomes, and interaction proteomes as well as dilute secretomes. The findings present a practical, sensitive and cost-effective proteomics sample preparation method.

## Abstract (precis)

The phenomenon of protein aggregation capture (PAC) on a wide range of different microparticles is described. Exploiting this mechanism enables generation of clean peptide mixtures from cell lines, tissues, and protein pulldowns for proteomics, phosphoproteomics, and secretomics analysis. The findings vastly increase the accessibility of the method that may ultimately lead to low cost and automated proteomics workflows.

## Graphical Abstract

**Figure:**
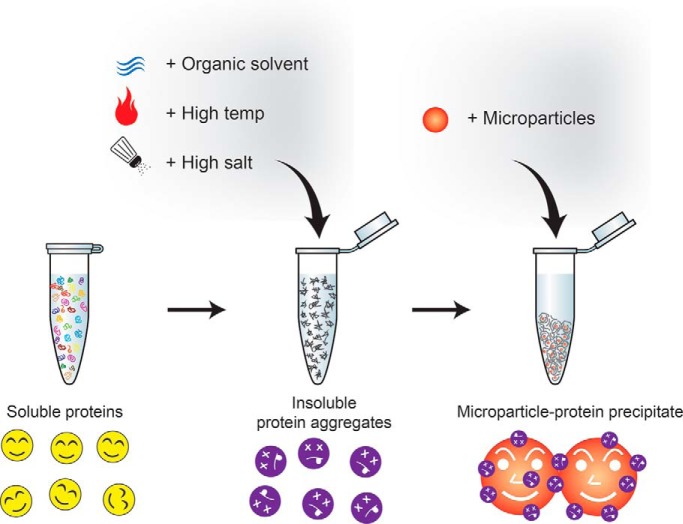

**Highlights**

- Insoluble protein aggregates preferentially precipitate on microparticles.
- The protein aggregation capture (PAC) occurs irrespective of microparticle surface chemistry.
- This process can be exploited for multi-purpose proteomics sample preparation.
- Facilitates potential for low cost, efficient and high-sensitivity proteomics workflows.

Dedicated sample preparation for shotgun proteomics is essential for removing impurities and interfering species which may affect peptide chromatography, ionization during the electrospray process, and sequencing by mass spectrometers. To represent the *in vivo* state of the global proteome including membrane-bound proteins, it is of high importance to ensure complete lysis of cells and tissues before protease digestion. This typically requires strong detergents that are difficult to remove afterward, however crucial to avoid signal interference during MS analysis. Considerable developments have been made based on a variety of different biochemical principles which use filters, traps, or protein precipitation techniques which address different sample types (1–3). However, a primary challenge remaining is the development of a universal sample preparation method that has the potential to scale across different sample amounts, which typically range from ng to mg of starting material. Moreover, such a method needs to be compatible with different lysis buffers, biological material (*i.e.* cell lines, tissues), robust, reproducible, cost effective, and perhaps above all; practical. Although several methods have been developed to individually address different proteomics sample preparation challenges, a simple solution spanning all sample types remains elusive. Here we report a mechanism, termed protein aggregation capture (PAC)^1^, which uses the phenomenon of nonspecifically immobilizing precipitated and aggregated proteins on any type of sub-micron particles irrespective of their surface chemistry. We explore the fundamental process underlying this phenomenon behind methods such as SP3 and determine the optimal parameters leading to effective sample preparation for shotgun proteomics analysis by mass spectrometry of different sample types. Our developments demonstrate the potential for low cost, simple, robust and sensitive sample preparation procedures for proteomics analysis, which can be easily implemented in any setting with great potential for full automation.

## EXPERIMENTAL PROCEDURES

### 

#### 

##### Reagents

Chemicals were purchased from Sigma-Aldrich (Søborg, Denmark) unless otherwise specified. 1 μm diameter Sera-mag carboxyl magnetic beads (cas # 45152105050250 and cas # 65152105050250) were purchased from GE-Healthcare (Brøndby, Denmark). 0.5 μm diameter SIMAG-Sulfon (cas # 1202), SiMAG-Q (cas # 1206), and SiMAG-Octadecyl (cas # 1301) magnetic beads were all purchased from Chemicell GmbH (Berlin, Germany). 5–10 μm average diameter HILIC, TiO2, and Ti-IMAC magnetic beads were purchased from ReSyn Biosciences (Edenvale, Gauteng, South Africa). Carbonyl-iron powder with 5–9 μm diameter grain size was purchased from Sigma-Aldrich (cas # 44890).

##### Cell Culture

Human bone osteosarcoma epithelial (U2OS) and human epithelial cervix carcinoma (HeLa) adherent cells were grown in DMEM media (Gibco, Thermo Fisher Scientific, Waltham, Massachusetts) supplemented with fetal bovine serum (Gibco) at 10% final. The media also contained penicillin (Invitrogen, Thermo Fisher Scientific) at 50 U/ml and streptomycin (Invitrogen) at 100 μg/ml. Cells were grown in a humidified incubator at 37 °C with 5% CO_2_. In all cases, cells were grown to 80–90% confluency before harvesting with different lysis buffers in Nunc petridishes (100 or 150 mm diameter).

To generate stably expressing GFP-TTP cells under a doxycycline inducible promoter, ZFP36/TTP was gateway cloned into a pCDNA4/TO/GFP expression vector by gateway cloning (Thermo Fisher Scientific), and co-transfected with pcDNA6/TR (Thermo Fisher Scientific) into U2OS cells. Cells were selected with zeocin and blasticidin for 14 days, after which individual clones were picked and screened for GFP-TTP expression. For SILAC labeling, cells were cultured in media containing either l-arginine and l-lysine (Light), l-arginine [^13^C6] and l-lysine [^2^H4] (Medium) or l-arginine [^13^C6-^15^N4] and l-lysine [^13^C6-^15^N2] (Heavy; Cambridge Isotope Laboratories, Tewksbury, Massachusetts).

RAW264.7 macrophage cells were derived from *Mus musculus* and grown in 10% in DMEM media with 10% FBS in 150 mm diameter Nunc petridishes. The media was removed, and cells were washed with PBS before addition of phenol-red free DMEM media without serum, penicillin, and streptomycin. Cells were stimulated with lipopolysaccharids (LPS) with 1 μg/ml for 4 h. Four hundred microliters of the media was removed and processed for secretome analysis and filtered through 0.22 μm filter (Sartorius #16532) before further processing.

##### Cell Lysis and Sample Preparation

Cells lysis as presented in this study was performed with either one of the three buffers: (1) 6 m guanidine hydrochloride in 100 mm tris hydrochloride (Life technologies, Carlsbad, California) at pH 8.5, (2) 1% SDS in 100 mm 100 mm Tris Hydrochloride (pH 8.5) or (3) 0.1% NP-40 in 1× phosphate buffered saline solution (pH 7.4) containing β-glycerol phosphate (50 mm), sodium orthovanadate (10 mm), and protease inhibitor mixture (Roche, Basel, Switzerland). In all cases, supernatant from adherent cell plates was removed and the cells were rinsed with ice cold 1× PBS before the addition of the lysis buffer.

Guanidine hydrochloride buffer was pre-heated to 99 °C before the addition to the cell plates. After the addition of guanidine or SDS buffer, cells were manually collected and heated at 99 °C for 10 min followed by sonication using a probe to shear RNA and DNA. Proteins were immediately reduced and alkylated with the 10 mm tris(2-carboxyethyl)phosphine (TCEP) and 11 mm 2-chloroacetamide (CAA) for samples lysed with guanidine hydrochloride or SDS lysis buffer before further processing. For cells lysed using 0.1% NP-40 buffer, 1 μl of benzonase (≥250U/ul) was added to the lysis solution for 1 h on ice. The lysis solution was centrifuged at 5000 × *g* for 10 min and the supernatant was transferred to a new tube.

GFP-TTP immunoprecipitations were performed using GFP-Trap magnetic agarose beads (Chromotek, Martinsried, Germany) according to manufacturer's instructions. Cell lysis and immunoprecipitations were carried out using low salt EBC lysis buffer (150 mm NaCl; 50 mm TRIS pH 7.5; 1 mm EDTA; 0,5% NP-40) for 1 h followed by 5 washes with the same buffer. Proteins were eluted by boiling in 2% SDS, 10% glycerol, 5% 2-mercaptoethanol, 0.1 m Tris-HCl (pH 6.8).

##### On-bead Protein Aggregation

Aggregation was induced by the addition of acetonitrile (unless stated otherwise) and magnetic microparticles were added to solution followed by mixing to uniformly mix the bead solution. The solution was allowed to settle for 10 min and beads were separated using a magnet for 60 s. Magnetic microspheres were retained by magnet and the supernatant was removed by vacuum suction. In the case of analysis by protein gel electrophoresis (SDS-PAGE), the supernatant was transferred to new tubes. Beads were washed using acetonitrile once followed by one wash with 70% ethanol. See extended methods for experiment specific protocols. Samples and washes were prepared for analysis by protein gel electrophoresis (SDS-PAGE) by the addition of 4× LDS sample buffer (Thermo Fisher Scientific) to 1× final, and DTT (100 mm). Samples were heated for 10 min at 80 °C. For eluting bead bound protein aggregates, LDS buffer (containing DTT) was added to bead containing solutions and the mixture was heated for 10 min at 80 °C. Heated beads in LDS buffer were separated by magnet and the supernatant was analyzed by SDS-PAGE or transferred to a new tube and stored at −20 °C until SDS-PAGE analysis. Samples were loaded on NuPAGE 4–12% Bis-Tris protein gel (Thermo Fisher Scientific) and ran with 200 volts for 40 min. Gels were stained for 15 min using instant Blue (Expedeon, San Diego, California) and destained overnight with Milli-Q water and scanned on EPSON V750 PRO.

##### Lys-C and Trypsin Digestion and Peptide Cleanup

Proteins were aggregated on microspheres and washed as described above. For on-bead digestion, 50 mm HEPES buffer (pH 8.5) was added to submerge microspheres. Proteins were reduced and alkylated with the 5 mm tris(2-carboxyethyl)phosphine (TCEP) and 5.5 mm 2-chloroacetamide (CAA) for 30 min if not treated immediately after lysis. Lys-c (Wako Chemicals) was added at ratio of 1:200 (to protein) and allowed to react for 1 h at 37 °C followed by the addition of trypsin at a ratio of 1:100 (unless specified otherwise). Trypsin digestion was allowed to occur overnight at 37 °C. Beads were separated by magnet and the supernatant was transferred to new tube and acidified.

In-solution digestion with guanidine hydrochloride buffer was carried out under similar reduction and alkylation conditions. Lys-c was added to solution and allowed to react for 1 h at 37 °C. The concentration of guanidine hydrochloride concentration was reduced to >1 M before the addition of trypsin for overnight digestion. Solution was acidified by with 1% trifluoroacetic acid (TFA) and centrifuged for 5 min at 5000 × *g* and the supernatant transferred to new tubes.

Peptide mixtures were clarified with solid phase extraction. Briefly, hydrophobic C18 sep-pak (Waters Corporation, Taastrup, Denmark) were prepared by washing with acetonitrile and 0.1% TFA, followed by loading of the acidified peptide mixtures by gravity. Sep-paks were washed with 0.1% TFA and peptides were eluted using 50% Acetonitrile (0.05% TFA). Organic solvent was evaporated and peptides concentrated using a speedvac before MS analysis.

##### Protein Extraction from Mouse Skeletal Tissue

We used skeletal muscle which were isolated for previously published study (See extended methods Schönke *et al.*). Frozen gastrocnemius muscles were crushed using mortar and pestle. Powdered muscle was homogenized using Ultra Turrax T8 homogenizer (IKA Labortechnik, Staufen im Breisgau, Germany) in 4% SDS buffer (100 mm Tris-HCl, pH 7.4). Protein lysates were boiled at 100 °C for 5 mins. Lysates were sonicated using a tip and centrifuged at 16,000 × *g* for 10 mins followed by reduction and alkylation as described above, the supernatant was then processed using FASP or PAC or frozen until further analysis.

##### Filter-aided Sample Preparation (FASP)

Urea powder was added to 400 μl of filtered cell culture supernatant for a final 2 m concentration, and pH for digestion adjusted with 40 μl Tris 1 m pH 8.5. FASP protocol was adapted from as previously described (1). Samples were heated for 10 min at 56 °C and centrifuged (7000 × *g*, 10 min). Following centrifugation steps were performed applying the same conditions. Ultracel-30 membrane filters (#MRCF0R030, Millipore, Burlington, Massachusetts) were cleaned with 10% acetonitrile and 15% methanol, filters were centrifuged and equilibrated with 200 μl urea buffer (2 m, 0.1 m Tris, pH8.5), and centrifuged again. Samples were added into the filters and the filters were centrifuged and washed two times with urea buffer. Reduction was performed by 1 μl of 0.5 m TCEP in 100 μl urea buffer. The device was centrifuged, and alkylation was performed by 1 μl of 550 mm CAA in 100 μl urea buffer for 30 min in the dark. Filters were centrifuged and 200 μl urea buffer was added before another centrifugation. Subsequently, 4 μl of 0.5 μg/μl lys-c in 40 μl urea buffer was added for 3h at 37 °C with gentle orbital shaking. 4 μl of 0.5 μg/μl trypsin was added for an overnight digestion in the wet chamber at 37 °C with gentle orbital shaking. 1.5 ml eppendorf tubes were cleaned with absolute methanol and air dried, before inserting the filter device, which was then centrifuged. Subsequently 40 μl of milli-Q water was added followed by centrifugation. The enzymatic digestion was stopped by acidifying the sample to pH < 2.5 with TFA. StageTipping was performed right after.

##### In-solution Urea Digestion Sample Preparation

All following chemicals have the same references and concentrations as in the FASP sample preparation. Urea powder was added to 400 μl of filtered (0.22 uM) cell culture supernatant for a final 2 m concentration, and pH for digestion adjusted with 40 μl Tris 1 m pH8.5. 100% v/v. TCEP was added and the tubes were incubated for 30 min. Subsequently, samples were incubated with CAA for 20 min in the dark. The digestion step included addition of lys-c, incubation during 3h, followed by the addition trypsin (0.5 μg/μl), and incubation overnight at room temperature. The enzymatic digestion was stopped by acidifying the sample to pH < 2.5 with TFA. Samples were desalted and concentrated using Stage-Tips.

##### In-gel Digestion

In-gel protein digestion and downstream processing was performed as described earlier (Lundby and Olsen 2011, see references in extended supplementary methods).

##### Enrichment of Phosphorylated Peptides

Adherent HeLa cells were grown as described above. Cells were washed and serum starved (DMEM without FBS) for 4 h followed by 10 min stimulation with FBS (10%). Cells were rapidly washed and lysed using guanidine hydrochloride buffer as described above. Protein concentration was estimated using tryptophan assay. Lys-C and trypsin digestion was carried out as described above. Peptides were clarified using SPE as described above with the exception that the peptide mixture was not concentrated using a speedvac. Small aliquat representing 5% was removed for determining peptide concentration using nanodrop which was estimated to roughly 200 μg.

Ultra high phosphopeptide enrichment efficiency was achieved using Ti-IMAC magnetic beads (ReSyn Biosciences) with slight modification to the manufacturer protocol (see extended supplementary methods). Phosphopeptide containing solution were loaded onto C18 STAGE-tips where the phosphopeptides were loaded and washed. The STAGE-tips were stored at 4 °C until elution and analysis by MS.

##### Mass Spectrometry and Liquid Chromatography

Samples were injected on a 15 cm nanocolumn (75 μm inner diameter) packed with 1.9 μm C_18_ beads (Dr. Maisch GmbH, Entringen, Germany) using an Easy-LC 1200 (Thermo Fisher Scientific). Peptides were separated and eluted from the column with an increasing gradient of buffer B (80% acetonitrile, 0.1% formic acid) at a flow rate of 250 nL/minute.

All samples were analyzed on a Q-Exactive HF-X (Thermo Fisher Scientific) mass spectrometer coupled to EASY-nLC 1200. Except for two replicates of in-gel TTP pulldown and one replicate from PAC TTP pulldown experiments were analyzed on a Lumos (Thermo Fisher Scientific) mass spectrometer with similar scan settings. The mass spectrometer was operated in positive mode with TopN method.

##### Data Analysis

All mass spectrometric data are available via ProteomeXchange with identifier PXD011677. Raw files generated from LC/MS/MS experiments were analyzed using MaxQuant (1.6.1.1) software (Cox and Mann 2008). Samples generated from human cell lines (HeLa and U2OS) were searched against the reviewed Swiss-Prot human proteome (proteome ID: UP000005640, release date March 2018) with 21006 entries. Samples generated from mouse cell lines (Raw264.7) and tissue were searched against the *Mus musculus* reviewed Swiss-Prot proteome (proteome ID: UP000000589, release date October 2018) with 22325 entries. The protease specificity was set to “Trypsin/P” with maximum number of missed cleavages set to 2 with the exception for the analysis of protease digestion efficiency experiment, where it was set to “semi-tryptic” search. All searches were performed with carbamidomethyl of cysteines as a fixed modification whereas methionine oxidation and protein n-terminus acetylation were set as variable. Phosphorylation of serine, threonine, and tyrosine were set as variable modification for analysis of phosphopeptide enriched samples using Ti-IMAC. Maximum number of modifications was set to 5 for all analysis. Mass tolerance of 20 parts per million (ppm) was set to the first search of precursor ions followed by 4.5 ppm for main search after mass calibration. 20 ppm mass tolerance was set for fragment ion series. Minimum peptide length of 7 amino acids was required for all identifications and modified peptides required a minimum Andromeda score of 40 be considered for identification. A false discovery rate (FDR) of 1% was used for peptide spectral matches, peptides, and proteins. Proteins had to be identified by minimum of 2 peptides to be counted. Match between runs feature was used only for the analysis of phosphopeptide enriched samples.

##### Experimental Design and Statistical Rationale

Number of replicates were denoted by “*n* = x” for all results where statistical analysis was performed and marked in the figures. Figure Legends provided further clarification of statistical tests and criteria for determining significance in each case. Analysis of protease efficiency were performed in duplicates for the two digestion methods (in solution *versus* PAC) with 25 different protease conditions for each, resulting in the analysis of 100 samples. One replicate from PAC digestion with no Lys-C and Trypsin at 1:50 ratio was discarded because of experimental error. Same biological source was used to limit the variation only to the sample preparation methods. For phosphoproteomics analysis (as presented in Fig. 3*B*, 3*C*, 3*D*), same HeLa protein extract was used to limit the variation to the sample preparation conditions. The protein extract was equally aliquated 8 times for quadruplicate analysis of digestion methods (in solution *versus* PAC) leading to 8 different samples. Each sample was independently prepared (in solution or PAC) followed by independent enrichment of phosphopeptides from each sample after protease digestion. Skeletal tissue protein extract was aliquated 6 times for triplicate analysis of peptide recovery and proteomics analysis between FASP and PAC. Each replicate was prepared and analyzed separately. SILAC analysis of ZFP36 (Tristetraprolin or TTP) interactors was performed in quadruplicates by growing cells in light, medium, and heavy states in 4 different cell culture plates (for a total of 12 different plates) and mixed to produce 4 separate samples from which ZFP36 interactors were determined. Elution from GFP-Trap beads were evenly split into half for either in-gel or PAC analysis. Quantile normalization was used to synchronize protein intensities and SILAC ratio distributions across replicates and experiments (see extended methods, Amaratunga *et al.* and Bolstad *et al.)*. Secretome analysis of RAW264.7 macrophage cells was performed in quadruplicates by growing the cells in 4 separate cell culture Petri dishes. The supernatant from each replicate was prepared independently for proteomics analysis by FASP, in solution, or PAC method.

## RESULTS

**Fig. 1.**
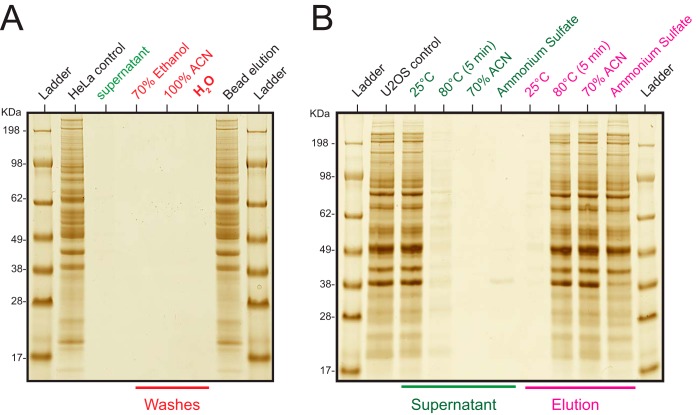
**Protein aggregation driven immobilization on microparticles.** *A*, The hypothesis of HILIC based bead interactions was tested by inducing bead - protein interaction on 20 μg of HeLa protein lysate (in 1% SDS) with the addition of acetonitrile (70% final concentration) and carboxyl coated microparticles (20 μg of Sera-mag beads) separated by magnet. The resulting supernatant was analyzed by SDS gel electrophoresis. Beads were sequentially washed three times with the indicated buffers. All washes were analyzed by SDS-PAGE for protein elution by beads including washes by milli-Q water and the lanes indicated in red. After bead washing, LDS buffer was added to the beads (see materials section) and analyzed. *B*, U2OS protein lysates (in 0.1% NP-40) were treated with different conditions as indicated in green. Carboxyl coated magnetic beads were added to the lysates after each treatment and the resulting supernatant analyzed by SDS-PAGE.

Our hypothesis was based on a series of reports and observations that lead us to conclude the mechanism of nonspecific aggregation, initially on magnetic beads with carboxyl group surface chemistries. Carboxyl coated magnetic beads have been reported for sensitive proteomics sample preparation as an alternative to other approaches such as FASP with limited starting material (4). The binding mechanism was attributed to hydrophilic interactions (HILIC)(5) with the carboxyl surface groups and the method was termed “SP3,” recent improvements of the protocol, such as pH control have rendered it more practical (6–8). As HILIC principles dictate preferential polar and ionic interactions under nonaqueous conditions, protein interaction to the carboxyl surface of the beads was hypothesized to be induced by the addition of acetonitrile to the protein lysate. However, we observed that stringent binding of proteins to the microspheres could not be completely reversed under aqueous conditions even with extended washing (Fig. 1*A*, supplemental Fig. S1*A*). Proteins however could be released in solubilization buffers such as lithium dodecyl sulfate (Fig. 1*A*). We therefore wondered whether protein immobilization could additionally be driven by aggregation of insoluble proteins on magnetic microspheres. To test this, we treated native protein lysates either by incubation at room temperature (25 °C) where proteins should stay in their native state, or induced aggregation by three different known mechanisms, these being organic solvent (acetonitrile; 70% final), high temperature (80 °C) for 5 min, or high salt (2.5 m ammonium sulfate), followed by the addition of magnetic carboxyl microspheres. Immobilization of aggregated and insoluble proteins was only observed under the three conditions known to induce aggregation, indicating that protein aggregation was essential to the underlying mechanism of protein capture (Fig. 1*B*). Importantly, the induced protein aggregation was very effective—especially using acetonitrile—as judged by the little protein amounts remaining in the supernatants.

**Fig. 2.**
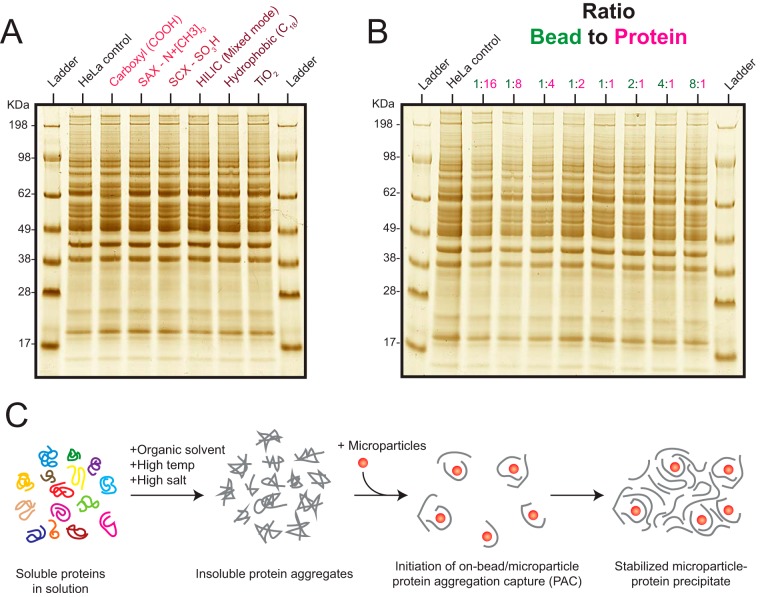
**Elucidating the mechanism of protein aggregation capture on microparticles.** *A*, Acetonitrile was added to HeLa lysate (in 1% SDS) to a final concentration of 70% and equal amounts of microparticles with different surface chemistries were added to the lysates and the supernatant removed. LDS buffer was added to the microparticles and the resulting supernatant analyzed by SDS-gel after removal by magnet. *B*, Aggregation of equal amount of HeLa lysate (20 μg at 0.25 μg/μl after addition of acetonitrile) was induced in a similar fashion as indicated above and carboxyl coated microparticles were added to the lysate at different amounts as indicated in the figure. The supernatant was removed, and the LDS buffer was added to the different samples and analyzed by SDS-PAGE after separation of microparticles by magnet. *C*, The hypothesized model for protein aggregation capture (PAC) on microparticles is illustrated based on the above observations.

We subsequently investigated the role of microsphere surface chemistry on protein immobilization and found no impact on protein aggregation irrespective of microsphere surface chemistry including those containing hydrophobic C_18_ surfaces (Fig. 2*A*). To rule out the role of the magnetic properties of the microspheres leading to immobilization, we tested protein aggregation on porous 3 μm C_18_ hydrophobic beads, which are typically used for packing reversed-phase nano-columns and found similar immobilization mechanisms (supplemental Fig. S1*B*). We further examined whether coated smooth surface microspheres were essential for protein immobilization by inducing protein aggregation (with acetonitrile) on fine iron powder microparticles (grain size 5–9 μm) and observed aggregation in a similar manner (supplemental Fig. S1*C*). However, because of the poor solubility of carbonyl powder (in water or water/organic solvent mix) the recovery was found to be less reproducible resulting in low protein aggregation especially as the volume of protein containing solutions were scaled higher (data not shown). Further, we tested the order addition of beads and solvent and found no noticeable effect of protein aggregation capture on beads (supplemental Fig. S1*D*). We next inquired whether protein aggregation on microspheres was a function of microparticle surface area by gauging protein aggregation at very low microsphere concentrations relative to a constant concentration of protein lysate at 0.25 μg/μl (Fig. 2*B*). Although very low amounts of beads were enough to aggregate proteins from solution (Fig. 2*B*), we found the structural integrity of visible protein-bead precipitate to be unstable when the bead to protein ratio was less than 1:4, leading to dispersion of small aggregated pieces in solution upon mild disruption. Conversely, solutions with low protein concentrations (<0.075 μg/μl) were found to require higher bead to protein ratios for efficient capture and recovery (supplemental Fig. S1*D*, S1*E*). These results indicate that solutions with higher protein concentrations can aggregate on minute amounts of microparticles, however lower protein concentration require relatively higher amounts of microparticles to effectively capture aggregated proteins. Collectively the data suggest that microparticle surface (irrespective of surface chemistry) acts as a nucleation site or carrier to induce a immobilization cascade of insoluble protein aggregates, which ultimately serve to tightly maintain a microparticle-protein structure (Fig. 2*C*).

**Fig. 3.**
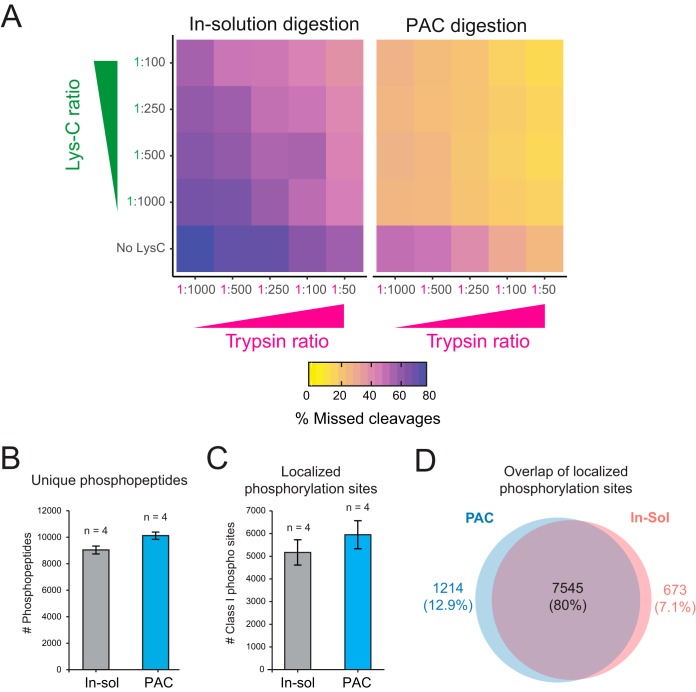
**Effects of protease digestion and post-translation modification (PTM) analysis of proteins immobilized on microparticles.** *A*, Average (based on duplicates) percentage of peptides containing missed cleavages at arginine or lysine after digestion with Trypsin and Lys-c proteases in different combinations and ratios are displayed by heatmap. Missed cleavage rates were investigated on lysates prepared by protein aggregation on microspheres or in-solution digestion. *B*, Average number of unique phosphopeptide variants were counted (after removal of contaminant or reverse hits as defined by MaxQuant analysis) for the different experiments. Phosphopeptides were tallied after enrichment from lysates prepared with in-solution or PAC digestion. *C*, Average number of phosphorylation sites with high localization probabilities (as defined by site localization probability ≥0.75) are presented between the two different methods. *D*, Overlap of localized phosphorylation sites (localization probably ≥0.75) between the two experiments. The site had to be identified in two of the four replicates in both experiments for it to be valid.

We assessed the impact on trypsin digestion efficiency of immobilized proteins on carboxyl coated microparticles and compared it to a commonly-used chaotropic agent based in-solution digestion protocol (9). Analyzing all samples by single shot nanoflow liquid chromatography tandem mass spectrometry (LC-MS/MS) we found significantly reduced number of missed tryptic cleavages between the two methods at different Lys-C and Trypsin ratios (Fig. 3*A*, supplemental Fig. S2*A*, supplemental Table S1). These findings imply the possibility to considerably reduce proteomics sample preparation costs as proteases typically constitute one of the largest expense of the workflow before MS analysis. As previously determined by missed cleavages across different digestion protocols (10), our results indicate that 10–20x reduction in Trypsin and Lys-C usage (1:500–1:1000 ratio) leads to comparable missed cleavage rates (below 30%) to the standard 1:50 trypsin-to-protein ratio used in most proteomics studies (Fig. 3*A*, supplemental Fig. 2*A*).

Post-translational modifications (PTMs) such as site-specific phosphorylation can rapidly modulate the function of proteins by changing their enzymatic activity, subcellular localization, turnover, and interaction partners (11). It is therefore important to develop proteomics methods that enable global analysis of phosphoproteomes in a robust, reproducible and sensitive manner. We examined whether the analysis of protein phosphorylation status is affected by the on-bead protein aggregation capture workflow on serum stimulated HeLa cells. Phosphopeptides were enriched by magnetic Ti-IMAC beads and the eluates analyzed by LC-MS/MS in turn. The results demonstrate no impact in the number of identified phosphorylated peptide variants and phosphorylation sites (Fig. 3*B*, 3*C*, supplemental Table S2). Importantly, >1000 more phosphorylated peptides and 779 localized sites were identified on average using microsphere protein aggregation followed by protease digestion compared with standard in-solution digestion. Moreover, high degree of overlap for localized sites was found between the two methods (Fig. 3*C*) and no bias in the phosphopeptide enrichment was observed between the two experiments as we achieved an enrichment efficiency >99% for all replicates (supplemental Fig. S2*B*). Surprisingly, we did not observe a major difference between missed cleavage rates for phosphopeptides between the two methods (supplemental Fig. S2*C*).

**Fig. 4.**
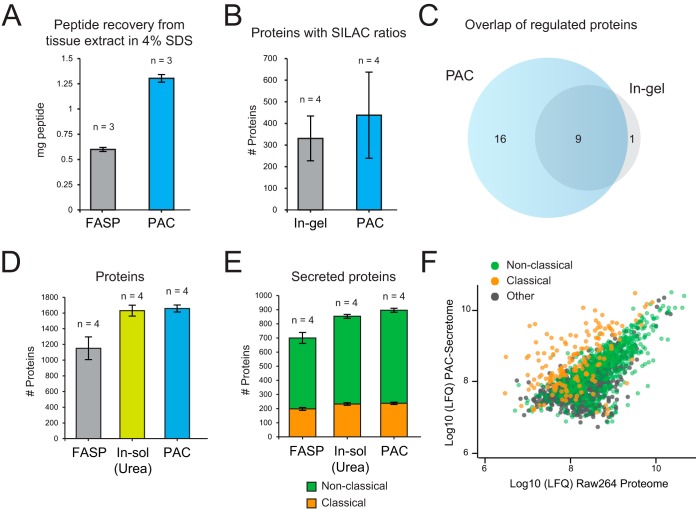
**Exploring the boundaries of PAC for proteomics analysis of different sample types.** *A*, Average peptide recovery after protease digestion of equal amounts (∼1.8 mg) of mouse skeletal tissue prepared using the FASP or PAC protocol measured by nanodrop absorbance at 280/260 nm. *B*, The average number of proteins with SILAC ratios were counted between the two different experiments after removal of contaminating proteins and reveres hits. *C*, Overlap of statistically regulated protein between two preparation methods as determined by *t* test with permutation-based FDR of 0.05 with 250 randomizations and s0 of 0.1 (supplemental Table 4). *D*, Number of proteins containing LFQ intensities from the different secretome workflows. *E*, Number of secreted proteins between the different experiments (see Methods). *F*, Median Log10 transformed LFQ intensities of the proteins identified in PAC are plotted against those identified in the base proteome of Raw264.7 cells. Classical and nonclassical secreted proteins are highlighted. *Error bars represent standard deviation in all cases.

We next assessed the potential of using magnetic microparticles for proteomics analysis of organs and tissues. This can be particularly challenging as it often requires harsh solubilization buffers for efficient protein extraction from hard and soft tissues. To test aggregation on microparticles we used skeletal muscle tissue samples from *Mus musculus*. After homogenization and solubilization in 4% SDS lysis buffer, we examined protein recovery and digestion by using either the established filter-aided sample preparation (FASP) protocol or via aggregation of proteins on magnetic microspheres with sulfonic acid surface chemistry. Significantly higher peptide recovery (>2 fold) after Lys-C/Trypsin digestion was observed using microspheres from initial starting material of 1.8 mg (as determined by tryptophan assay) (12) per replicate (Fig. 4*A*). Incidentally, sample preparation with magnetic microspheres resulted in a cleaner peptide mixture as no polymer peaks were observed in the mass spectrometry analysis, leading to higher number of identified proteins and unique peptides in a single shot LC-MS/MS analysis (supplemental Fig. S2*D*, S2*E*, supplemental Table S3).

Proteins rarely operate alone in the cell and their function is usually dependent on the protein complexes they are part of (13). One potential application of magnetic microparticles is for the rapid analysis of protein-protein interactions (PPIs). Antibody-based pulldown in combination with mass spectrometry is a popular approach for elucidating PPIs. We inquired whether a simplified microparticle based methods such as SP3 for analyzing PPIs provides depth of coverage comparable to standard in-gel protocols. To test this, we used a SILAC (14) based setup for determining interactors of ZFP36 (Tristetraprolin or TTP), an RNA binding protein (supplemental Fig. S3*A*). On average we identified 100 more proteins (438) when eluted proteins were aggregated on beads (with HILIC surface groups) based on single shot MS analysis compared with a conventional in-gel digestion workflow (330) with 5 fractions per replicate (Fig. 4*B*, supplemental Fig. S3*B*, supplemental Table S4). Crucially, we found good overlap between the two groups for IL-1b regulated TTP-interactors (Fig. 4*C*, supplemental Table S4). We identified known interactors of TTP upon IL-1b stimulation such as 14-3-3 subunits and RNA-binding factors which regulate stability such as UPF1 and PABC1/4 (15) (supplemental Fig. S3*C*), as well as potential novel interactors which displayed interesting interaction dynamics with TTP upon IL-1b stimulation (supplemental Fig. S3*D*).

MS-based analysis of cellular secretome holds enormous promise for the investigation of cellular communication. However, the analysis of cellular secretome presents several challenges as proteins are usually secreted in low concentrations making their detection in culture media (which are rich in salts and other compounds) difficult (16). We sought to determine the applicability of microparticles for enriching secreted proteins in the background of cell culture media contaminants. To this end we benchmarked urea (in-solution) and filter (FASP) based methods as reported previously (17, 18), against PAC on microparticles for secreted proteins. As described above (supplemental Fig. S1*E*, S1*F*), high concentration of microspheres (>300 μg/ml) were required to provide enough surface area for immobilization of dilute aggregated proteins. Protein aggregation on microspheres consistently identified the largest number of proteins and unique peptides resulting in the highest sequence coverage (Fig. 4*D*, supplemental Fig. S4*A*, S4*B*, supplemental Table S5). Although the low peptide recovery with FASP protocol led to the fewest number of protein identifications, the microsphere and FASP methods produced the cleanest peptide sample as determined by spectroscopy analysis (supplemental Fig. S4*C*, S4*D*). Using previously described computational workflow to predict potentially secreted proteins (18) https://paperpile.com/c/bj70VS/UXgB, we found majority (>50%) of the detected proteins have been previously characterized as secreted proteins through the classical secretory pathway (via signal peptide) or other nonconventional pathways (Fig. 4*E*) (19). Proteins secreted through the classical pathway were found at higher abundance relative in the media (Fig. 4*F*). This includes low abundant cytokines such as Csf3 and Cxcl10 which were only identified using by aggregating proteins on magnetic microbeads, demonstrating sensitivity of the protocol. Thus, microparticle aggregation can enable simultaneous quantification of 100s of secreted proteins in the background of complex cell culture media.

## DISCUSSION

We have described the protein aggregation capture mechanism behind microsphere based protein immobilization. Understanding the mechanism of PAC in detail has led to optimized protocols that outperform competing methods for preparing samples for proteomic analysis of tissue, enriched subproteomes such as phosphoproteomes, low abundant immunoprecipitated and secreted proteins. The rates of missed cleavages were low (<30%) when proteins aggregated on-beads were digested with Lys-c/Trypsin. Although the rates of missed cleavages were significantly higher for phosphopeptides (66–67%) using the PAC protocol, we saw no difference in the rate using the other methods tested. This can be explained by the fact that phosphate groups are known to impair protease digestion efficiency (20, 21). In the secretome analyses reported here, serum-containing media was replaced with serum-free media before stimulation which avoided the usual challenge of large dynamic range of high amounts of albumin that can obstruct detection of secreted proteins. Alternatively, newly synthesized secreted proteins could be enriched by bio-orthogonal amino acid incorporation in combination with protein aggregation on beads (22). We demonstrate that PAC is scalable from very low to high starting amounts of material, which has advantages for reducing cost, simplicity, and time. The workflow is unbiased for downstream sequencing of peptides and would be compatible with alternative novel protein sequencing technologies such as those that use fluorophores labeling and sequencing of peptide mixtures (23). The PAC approach could potentially fail if samples contain certain components (such as acids) that could prevent efficient aggregation on microparticle surfaces. Other large biomolecules such as DNA/RNA can also co-precipitate with proteins if not adequately removed. We hope awareness of this mechanism will lead to further novel developments and applications. Future developments could use microparticle surface functional group specificities for peptide level enrichment/fractionation following nonspecific aggregation at the protein level.

## Data Availability

All mass spectrometric data are available via ProteomeXchange with identifier PXD011677. Annotated spectra of post-translationally modified peptides can be found using the MS-Viewer here (The search key for the saved data set is lj9dbrbqem): http://msviewer.ucsf.edu/prospector/cgi-bin/mssearch.cgi?report_title=MS-Viewer&search_key=lj9dbrbqem&search_name=msviewer.

## Supplementary Material

supplemental Table 4

Supplementary table 1

Supplementary table 2

Supplementary table 3

Supplementary table 4

Supplementary table 5

Extended methods

Supplementary figures and legends

## Footnotes

^1^ The abbreviations used are:

PAC: protein aggregation capture.
