# Supplementary material for: Protein Aggregation Capture on Microparticles Enables Multipurpose Proteomics Sample Preparation
Source: Mol Cell Proteomics. 2019 Mar 4;18(5):1027–35. doi: 10.1074/mcp.TIR118.001270 (PMC6495262; doi:10.1074/mcp.TIR118.001270)
Supplement: supplemental Table 4 [file TIR118.001270_index.html]

Supplement to Protein aggregation capture on microparticles enables multi-purpose proteomics sample preparation. | Molecular & Cellular Proteomics

## Supplemental Data

- Supplementary table 1 - This table contains missed cleavage rates for experiments performed using in-solution digestion or protein aggregation on carboxyl beads using acetonitrile.
- Supplementary table 2 - This table contains results from the phosphoproteomics analysis. The table contains peptide level evidence produced from the MaxQuant output evidence.txt as well as phosphorylation sites table produced from the output Phospho (STY)Sites.txt
- Supplementary table 3 - Comparison of proteins identified from mus musculus skeletal muscle tissue after preparation with FASP or PAC. This table contains list of protein identifications produced from the MaxQuant output protein.txt as well as peptide level evidence table produced from the output evidence.txt
- Supplementary table 4 - This table contains data for proteins identified and SILAC ratios after pulldown of ZFP-36-GFP. The table contains list of protein identifications produced from the MaxQuant output protein.txt.
- Supplementary table 5 - This table contains list of protein identifications from secretome analysis of macrophage cells. The table contains list of proteins identifications produced from the MaxQuant output proteingroups.txt.
- Extended methods - This file contains detailed protocol for aggregation of proteins for different experiments. In-depth protocol for enrichment of phosphopeptides using magnetic Ti-IMAC beads is also included.
- Supplementary figures and legends - This file contains supplementary figures and figure legends.
