# Supplementary material for: Protein Aggregation Capture on Microparticles Enables Multipurpose Proteomics Sample Preparation
Source: Mol Cell Proteomics. 2019 Mar 4;18(5):1027–35. doi: 10.1074/mcp.TIR118.001270 (PMC6495262; doi:10.1074/mcp.TIR118.001270)
Supplement: Extended methods [file 142488_2_supp_292076_pnj48d.pdf]

EXTENDED SUPPLEMENTARY METHODS

Protein aggregation capture for SDS-PAGE analysis

Experiments were performed in a Thermo Fisher Scientific 96 well plates (#AB1300). DynaMag-96 Side Skirted Magnet separator (#12027) was utilized to separate magnetic particles. Cell lysates should be cleared of DNA/RNA by sonication or nucleases (such as benzonase) in order to ensure efficient on-bead protein precipitation.

1) Acetonitrile was added to protein extract lysates to reach a final concentration of 70%.

2) Beads were added to wells immediately afterwards and uniformly mixed.
   a) In experiments where no organic solvent was used, beads were mixed after addition of high concentration ammonium sulfate or temperature heating in a similar manner.

3) Solution was allowed to remain for 10 minute at room temperature.

4) 96 well plate was placed on a magnetic separator and beads were allowed to separate for 60 seconds.

5) Supernatant was transferred to new tubes and speedvac'd to remove organic solvent for SDS-PAGE analysis.
   a) Supernatant was discarded by vacuum suction if it was not to be analyzed by SDS-PAGE.

6) 100 µL of 70% ethanol was added to the wells containing magnetically separated beads and allowed to remain for 10 seconds.

7) 70% ethanol was transferred to new tubes and speedvac'd to remove organic solvent for SDS-PAGE analysis.
   a) 70% ethanol was discarded by vacuum suction if it was not to be analyzed by SDS-PAGE.

8) 100 µL acetonitrile of was added to the wells containing magnetically separated beads and allowed to remain for 10 seconds.

9) Acetonitrile was transferred to new tubes and speedvac'd to remove organic solvent for SDS-PAGE analysis.
   a) Acetonitrile was discarded by vacuum suction if it was not to be analyzed by SDS-PAGE.

10) 96 well plate containing beads were removed from magnet.

11) 20µL or 40µL (depending on if sample were to be analyzed by 12 well or 10 well NuPAGE 4-12% Bis-Tris protein gels respectively) of 1x LDS buffer containing 100mM DTT was added to the beads.

12) Dried supernatant and washes from acetonitrile and 70% ethanol were reconstituted in 1x LDS buffer and 100mM DTT final.

13) Samples were heated to 80°C for 10 minutes in a thermocycler (for 96 well plates).

14) Heated samples with beads were separated by magnet and transferred to new well or tubes.

15) Samples were analyzed by SDS-PAGE or stored at -20°C.

Protein aggregation capture for protein phosphorylation analysis

One limit of aggregating proteins on microparticles that we observed was low peptide recovery (after protease digestion) from protein lysates containing high concentration chaotropic salts such as guanidine hydrochloride (6M). We attributed this to phase separation upon addition of organic solvents such as acetonitrile in the aqueous sample buffer. This reduced protein aggregation as proteins were remained soluble in the aqueous phase containing guanidine hydrochloride. Phase separation could be however ameliorated by diluting the concentration of the guanidine buffer with water prior to the addition of organic solvent. This was determined by peptide recovery following on-bead trypsin digestion (See figure S1 below). Alternatively, organic solvents with higher water solubility could be also be utilized to prevent phase separation.

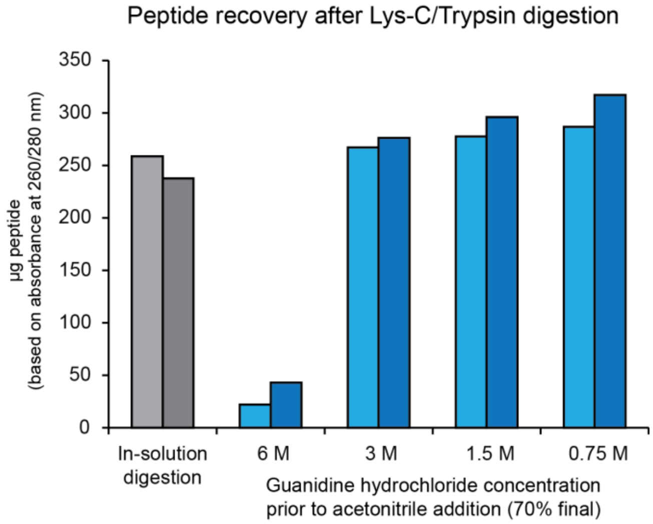

Figure S1. Recovery of peptides after lys-c/trypsin digestion using aggregation on microparticles after dilution of the guanidine-hydrochloride lysate with water at different concentrations.

On-bead aggregation followed by downstream protease digestion was carried out in 1.5ml Eppendorf Lo Bind 1.5ml tubes. Beads were separated on DynaMag-2 Magnet (#12321D).

1) 200 µg of HeLa lysate in 6M guanidine lysis buffer (100 mM Tris pH 8.5) was diluted 1x with milli-Q water.

2) Acetonitrile was added to obtain a final percentage of 70%.

3) 400 µg of magnetic beads were immediately added and mixed thoroughly in order to create a uniform solution.

4) Solution was allowed to sit for 10 minutes after which the slurry was mixed again and allowed to sit for another 10 minutes for a total of 20 minutes.

5) Tubes were placed on magnetic rack and allowed to separate for 30 seconds.

6) All subsequent washes were performed without removing the eppendorf tubes from the magnetic rack.

7) Supernatant was removed by vacuum suction via tip.

8) 1 ml of neat acetonitrile was added to the tubes and allowed to sit for 10 seconds and removed.

9) 1 ml of 70% ethanol was added to the tubes and allowed to sit for 10 seconds and removed.

10) Tubes were removed from the magnetic rack and 400 µL of digestion buffer (50mM HEPES, pH 8.5) was added to the tubes.

11) Dried beads which adhered to tube walls were manually displaced by tip until submerged in digestion buffer.

12) Digestion was carried out using lys-c and trypsin as described in Methods.

13) Samples were acidified to 0.5% TFA after overnight digestion.

14) Acidified tubes were placed on magnetic rack for 60 seconds and the supernatant was transferred to new tubes.

15) Supernatant in new tubes were put on a magnetic rack in order to remove residual beads and the supernatant was transferred again to new 1.5 ml tubes.

16) Supernatant was cleared by C18 SPE.

Protein aggregation capture for tissue proteome analysis

On-bead aggregation was carried out similarity as described above in 1.5ml Eppendorf Lo Bind 1.5ml tubes and beads separated using DynaMag-2 Magnet with the exception that dilution with milli-Q water was not necessary. However we find larger starting material leads to co-precipitation of DNA/RNA from tissue material despite extensive sonication and centrifugation steps, therefore additional measures must be taken to adequately remove residual precipitated DNA/RNA.

1) Add acetonitrile to the biological extract to final percentage of 70%.
   a) Samples in large volumes >500ul can either be split into smaller volumes in 1.5ml Eppendorf tubes or the aggregation can be performed in large 15ml or 50ml tubes.
   b) It must be noted that aggregation with acetonitrile in the large tubes can lead to protein aggregation forming on tube walls prior to the addition of magnetic particles.
   c) This can be circumvented by adding magnetic particles to the solution prior to the addition of organic solvent, adding dilute detergent to the solution, or using alternative organic solvents such as ethanol or isopropanol.

2) 400 µg of magnetic beads were immediately added and mixed thoroughly in order to create a uniform solution.
   a) Precipitation using acetonitrile can cause bead-protein precipitate to stick to the side of the tubes.
   b) Beads can be added prior to the addition of acetonitrile if excessive aggregation on tube walls is observed.
   c) In the case of bead/protein precipitating on tubes, washes and digestion can still be performed if caution is made to cover the beads in digestion buffer in order to prevent bead-proteins from drying out.

3) Solution was allowed to sit for 10 minutes after which the slurry was mixed again and allowed to sit for another 10 minutes for a total of 20 minutes.

4) Tubes were placed on magnetic rack and allowed to separate for 30 seconds.

5) All subsequent washes were performed without removing the eppendorf tubes from the magnetic rack.

6) Supernatant was removed by vacuum suction via tip.

7) Follow steps 8-15 as described above.

8) Centrifuge tubes for 10 minutes at 16,000 x G in order to remove residual DNA/RNA.

9) Transfer supernatant to new tubes and clear peptide supernatant using SPE.

10) After elution and concentration of peptides using speedvac, residual DNA/RNA precipitate can remain.

11) Centrifuge tubes again 10 minutes at 16,000 x G.

12) Transfer supernatant to new tubes and analyze by LC/MS/MS.

Enrichment of phosphorylated peptides

Ti-IMAC Beads were prepared according to manufacturer instructions (Resyn Biosciences) with slight modifications as described below. Enrichments were carried out in Eppendorf protein LoBind 96 well plates (#0030504119). The plates were mixed on a Heidolph Titramax 1000 96 well plate shaker (#544-12200-00). Beads were separated in the 96 well plate using a Thermo Fisher Scientific Ambion magnetic stand (#AM10027).

1) Loading buffer (80% acetonitrile, 1M glycolic acid, 5% TFA) was added to eluted peptide mixture at 1:1 volume ratio.

2) 200µg of Ti-IMAC beads (10µl) were added to 200µg peptide mixture (1:1 ratio) and the binding occurred at 1350 RPM on for 20 minutes.

3) Place 96 well plate on magnetic stand and allow separation for 20 seconds.

4) Carefully remove the supernatant using gel-loader tip connected to a vacuum.
    a) Do not disrupt the magnetically separated beads with the tip during suction.
    b) The magnetic beads should be displaced to the side of the well walls by magnet and not concentrated at the bottom of the well.
    c) Remove residual liquid in the wells down to the last drop in the bottom of the well.

5) Add 400 µl of loading buffer and place the plate on the plate shaker.

6) Mix for 1 minute at 1350 RPM.

7) Remove the supernatant as described in step 4.

8) Wash the beads with 400 µl of washing buffer 1 (80% acetonitrile, 1% TFA) for 2 minutes at 1350 RPM.

9) Remove the supernatant as described in step 4.

10) Repeat steps 8 and 9.

11) Wash the beads with 400 µl of washing buffer 2 (10% acetonitrile, 0.2% TFA) for 2 minutes at 1350 RPM.

12) Remove the supernatant as described in step 4.

13) Repeat steps 11 and 12.

14) Add 80 µl 1% ammonium hydroxide to the bead containing wells and mix for 20 minutes at 1350 RPM.

15) Place the 96 well plate on magnetic separator.

16) Transfer the supernatant to 1.5 ml Protein LoBind tubes (Eppendorf).

17) Repeat step 14 and 15.

18) Transfer the supernatant again to the same 1.5 ml Protein LoBind tubes.

19) Repeat step 14 and 15 for final elution.

20) Transfer the supernatant again to the same 1.5 ml Protein LoBind tubes for a final volume of 240 µl.

21) Add 60 µl of 10% formic acid.

22) Speedvac solution and reconstitute in 0.1% formic acid if supernatant is completely evaporated.

23) Desalt phosphopeptides using C18 STAGE-tips (or other material) and store at 4°C until elution and analysis by MS.

EXTENDED REFERENCES

Cox J, Mann M. 2008. *MaxQuant Enables High Peptide Identification Rates, Individualized P.p.b.-Range Mass Accuracies and Proteome-Wide Protein Quantification*. Nature Biotechnology 26 (12): 1367–72.

Tyanova S, Tikira T, Pavel S, Carlson A, Hein MY, Geiger T, Mann M, Cox J. 2016. *The Perseus Computational Platform for Comprehensive Analysis of (prote)omics Data*. Nature Methods 13 (9): 731.

Lundby A, Olsen JV. 2011. *GeLCMS for in-Depth Protein Characterization and Advanced Analysis of Proteomes.* Methods in Molecular Biology 753: 143–55.

Amaratunga D, Cabrera J. 2001. *Analysis of Data From Viral DNA Microchips.* Journal of the American Statistical Association 96, 1161–1170.

Bolstad BM, Irizarry RA, Astrand M, Speed TP. 2003. *A comparison of normalization methods for high density oligonucleotide array data based on bias and variance*. Bioinformatics 19, 185-193.

Schönke M, Björnholm M, Chibalin AV, Zierath JR, Deshmukh AS. 2018. *Proteomics Analysis of Skeletal Muscle from Leptin-Deficient ob/ob Mice Reveals Adaptive Remodeling of Metabolic Characteristics and Fiber Type Composition*. Proteomics 18, 1700375.
