# Supplementary material for: Protein Aggregation Capture on Microparticles Enables Multipurpose Proteomics Sample Preparation
Source: Mol Cell Proteomics. 2019 Mar 4;18(5):1027–35. doi: 10.1074/mcp.TIR118.001270 (PMC6495262; doi:10.1074/mcp.TIR118.001270)

Supplementary figures

Supplementary Figure 1

A) Acetonitrile was added to 20µg of HeLa lysate (in 1% SDS) at a final concentration of 70% and the supernatant including sequential washes (including a final 30 minute milli-Q water wash) were analyzed by SDS-PAGE. B) Protein aggregation on non-magnetic microparticles (3µm hydrophobic beads) was analyzed by SDS-PAGE of HeLa lysate aggregated by acetonitrile in a similar fashion. Beads were separated from supernatant by brief (30 second) centrifugation on a benchtop centrifuge. C) Aggregation of 20µg of HeLa lysate (in 1% SDS) on carbonyl iron powder was assessed. After inducing aggregation with acetonitrile, the magnetic powder was separated using a magnet the supernatant was analyzed by SDS-PAGE. Eluate was obtained by adding LDS buffer to the powder and analyzed (after heating at 80°C) by SDS-PAGE. D) Bead and solvent addition order was tested on 20µg of HeLa lysate (in 1% SDS) using 40µg of carboxyl coated magnetic beads. Magnetic beads were added before and after the addition of acetonitrile. Supernatant and elution (in LDS buffer) were analyzed using SDS-PAGE. E) Recovery of 10µg of HeLa lysate from 40µg of carboxyl coated microparticles were analyzed by SDS-PAGE after the lysate was diluted to decreasing concentration with addition of water and acetonitrile (70% final). F) The amount of microparticles were increased and the capture and elution of highly dilute HeLa lysate from the beads was analyzed by SDS-PAGE.

Supplementary Figure 2

A) Data from Figure 2A is presented in bar plots indicating the percentage of peptides containing missed cleavages at arginine or lysine after digestion with trypsin and lys-c proteases in different combinations and ratios. B) Phosphopeptide enrichment efficiency percentage is shown for all the experiments and replicates in the data presented in Figure 2B. Efficiency was determined by counting all peptides with or without phosphoryl modification (at serine, threonine, tyrosine) in each experiment

and after removal of all contaminating and reverse peptides from the evidence.txt output generated by MaxQuant analysis (see Methods). *Error bars represent standard deviation. C) Proteins identified with iBAQ intensities from MaxQuant ProteinGroups.txt output between the two experiments after single shot MS analysis. D) Unique peptides identified between the two experiments and replicates obtained from the evidence.txt MaxQuant output.

Supplementary Figure 3

A) SILAC experimental scheme for GFP-TTP pulldown after doxycyclin induction and/or IL-1b stimulation. TPP was expressed with an N-terminal GFP-tag (GFP-TTP) and the bait and interactors were enriched using commercial GFP-Trap beads. Expression TTP with an N-terminal GFP-tag (GFP-TTP) was induced by doxycycline and the interaction dynamics determined after IL-1b stimulation. B) In-gel scheme for generating 5 fractions for in-gel digestion. C) Median of quantile normalized intensities of the proteins were plotted against the median quantile normalized heavy over median (H/M) ratios for all the proteins identified after analysis of GFP-TTP pulldown eluates prepared using PAC. D) Volcano plot of the difference between TTP interacting proteins after 24 hour doxycyclin stimulation (M/L) vs doxycyclin and IL-1b and treated cells at the same time point (H/L) . *Error bars represent standard deviation.

Supplementary Figure 4

A) Unique peptides identified from the secretome of Raw264.7 cells using different workflows. Unique peptides were counted from the MaxQuant evidence.txt output after removal of duplicate modified sequences as well as reverse and contaminant peptides from each replicate. B) Average sequence coverage was obtained from the identified proteins in each replicate of the secretome workflows. C) Spectroscopy analysis of peptides concentrated and eluted using Stage-Tips after the different secretome workflows shows absorbance and contaminant values at the indicated wavelengths. D)

Spectroscopy analysis at different UV wavelengths for the different experiments and replicates is presented.

Supplementary Figure 1

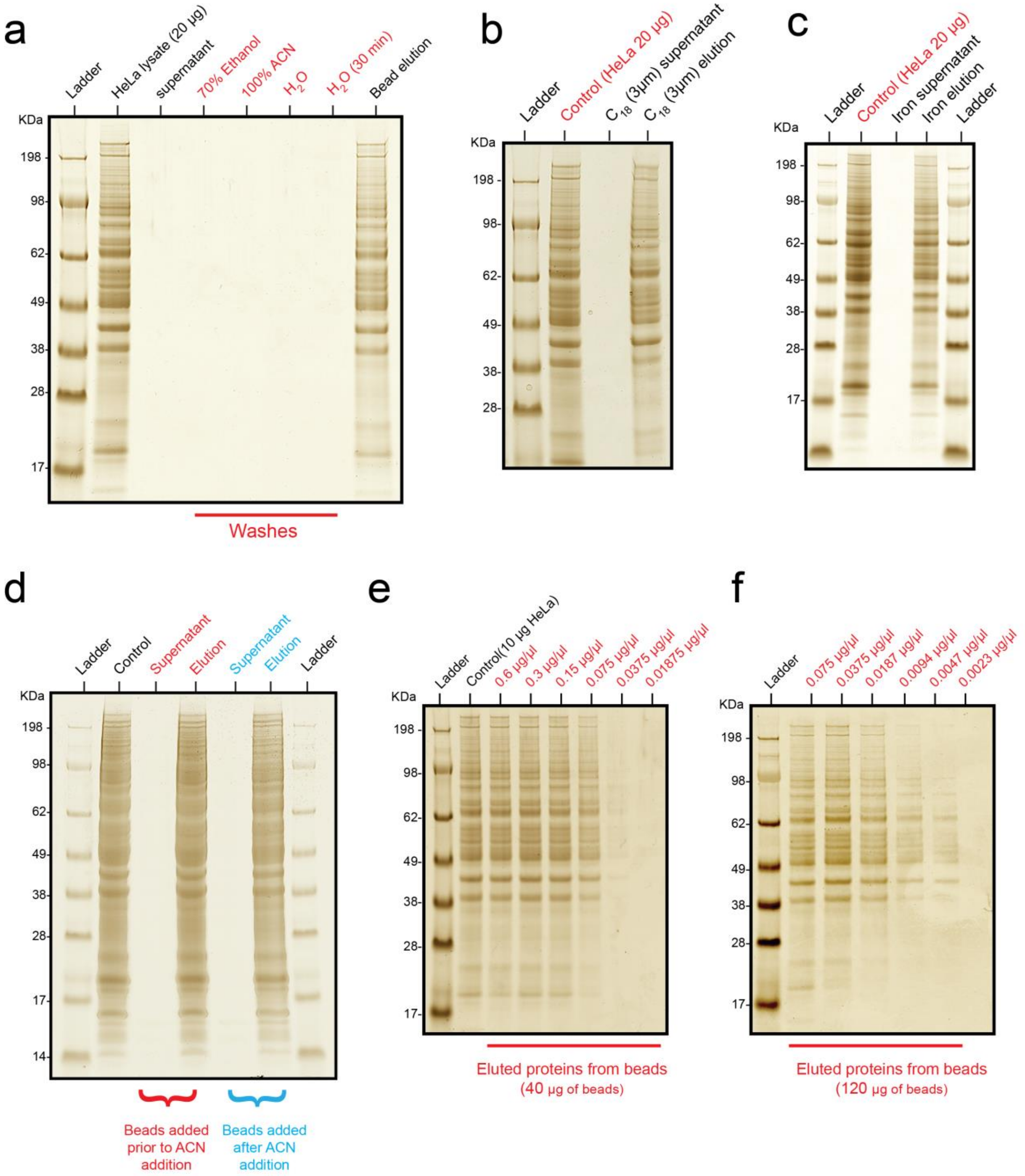

Supplementary Figure 2

a

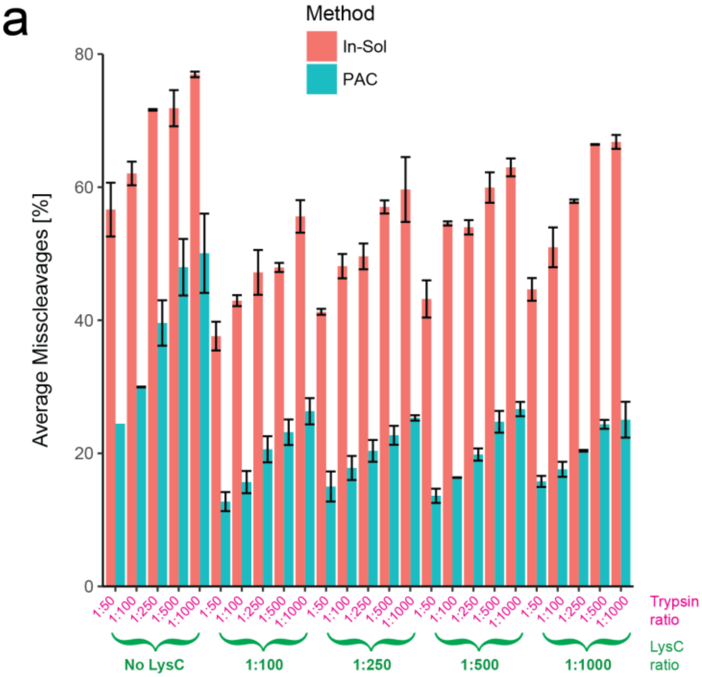

b

Phosphopeptide enrichment efficiency %

Sample	Phosho-peptides	All peptides	%
Gnd-HCl Rep 1	8680	8727	99.46
Gnd-HCl Rep 2	9308	9362	99.42
Gnd-HCl Rep 3	9264	9301	99.60
Gnd-HCl Rep 4	8915	8951	99.60
PAC Rep 1	10293	10333	99.61
PAC Rep 2	10049	10108	99.42
PAC Rep 3	10377	10416	99.63
PAC Rep 4	9764	9821	99.42

c

Phosphopeptide missed cleavage %

Sample	0	1	2	% Missed cleavages
Gnd-HCl Rep 1	2513	3460	2754	71.20
Gnd-HCl Rep 2	3050	3733	2579	67.42
Gnd-HCl Rep 3	3053	3686	2562	67.18
Gnd-HCl Rep 4	2564	3562	2825	71.36
PAC Rep 1	3385	4145	2803	67.24
PAC Rep 2	3538	4033	2537	65.00
PAC Rep 3	3392	4127	2897	67.43
PAC Rep 4	3223	3915	2683	67.18

d

Proteins identified

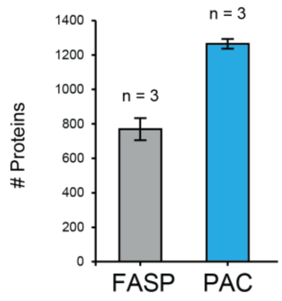

e

Unique peptides

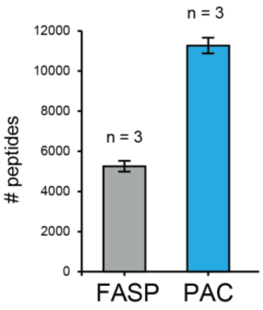

Supplementary Figure 3

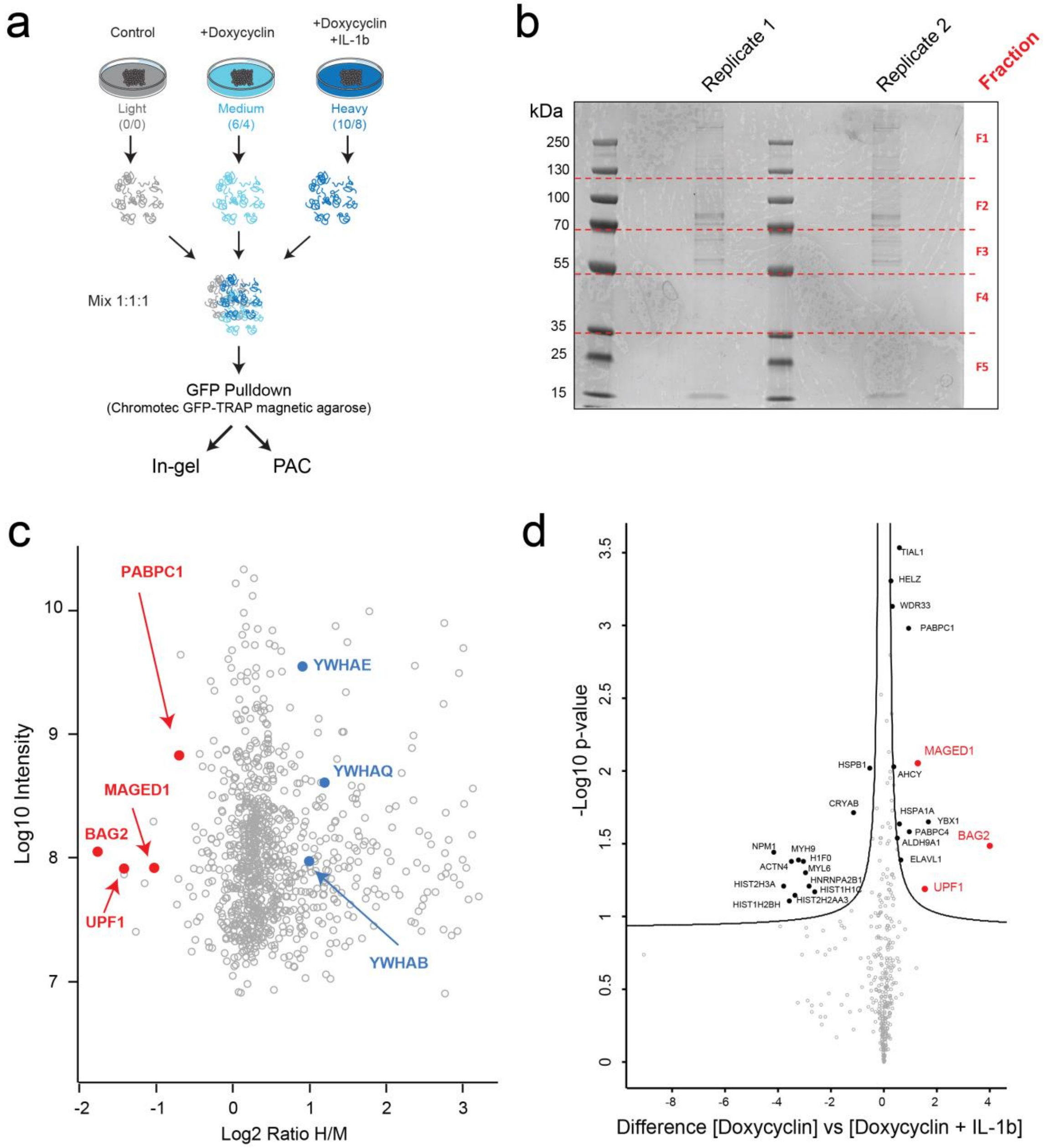

Supplementary Figure 4

a

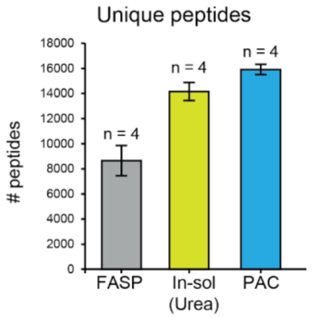

b

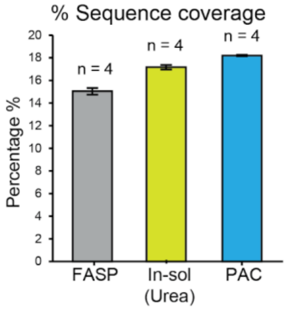

c

Sample Name	Protein(mg/mL)	A280	A260/A280	Sample Type	Baseline Correction (nm)	Baseline Absorbance	Corrected (mg/mL)	Corrected %CV	Impurity 1	Impurity 1 A280	Impurity 1 %CV
Urea Insol 1	8.251	8.251	0.897	1 Abs = 1mg / mL	340	2.392	7.55	4.36	Nucleic Acid	1.972	13.66
Urea Insol 2	7.812	7.812	0.89	1 Abs = 1mg / mL	340	2.697	7.273	4.07	Nucleic Acid	1.829	14.55
Urea Insol 3	6.571	6.571	0.906	1 Abs = 1mg / mL	340	2.098	6.081	3.89	Nucleic Acid	1.517	13.69
Urea Insol 4	7.795	7.795	0.91	1 Abs = 1mg / mL	340	8.19	7.009	4.01	Nucleic Acid	1.69	19.43
Urea Insol 5	7.786	7.786	0.893	1 Abs = 1mg / mL	340	2.503	7.199	3.96	Nucleic Acid	1.83	14.03
PAC 1	0.821	0.821	1.031	1 Abs = 1mg / mL	340	0.395					
PAC 2	0.726	0.726	1.018	1 Abs = 1mg / mL	340	0.413					
PAC 3	0.735	0.735	1.013	1 Abs = 1mg / mL	340	0.359					
PAC 4	0.778	0.778	1.015	1 Abs = 1mg / mL	340	0.43					
PAC 5	0.757	0.757	1.004	1 Abs = 1mg / mL	340	0.394					
FASP 1	0.14	0.14	1.071	1 Abs = 1mg / mL	340	0.045					
FASP 2	0.106	0.106	0.941	1 Abs = 1mg / mL	340	-0.19					
FASP 3	0.151	0.151	1.085	1 Abs = 1mg / mL	340	0.07					
FASP 4	0.162	0.162	0.979	1 Abs = 1mg / mL	340	0.037					
FASP 5	0.166	0.166	0.973	1 Abs = 1mg / mL	340	0.028					

d

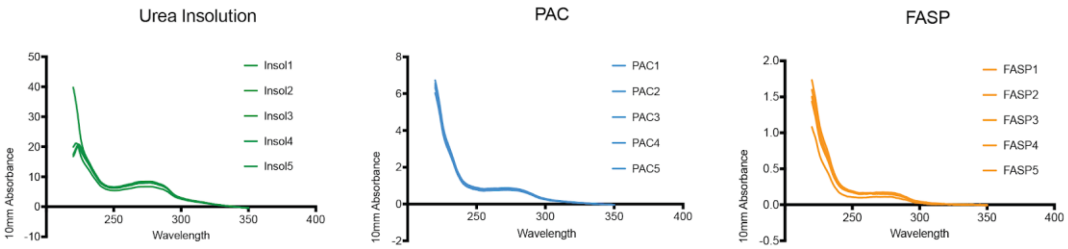

Supplement: Supplementary figures and legends [file 142488_2_supp_292079_pnj4bq.pdf]
